# Supplementary material for: Citroflavonoids as Promising Agents for Drug Discovery in Diabetes and Hypertension: A Systematic Review of Experimental Studies
Source: Molecules. 2022 Nov 16;27(22):7933. doi: 10.3390/molecules27227933 (PMC9696987; doi:10.3390/molecules27227933)
Supplement: Supplementary file 1 [file molecules-27-07933-s001.zip › Supplementary Materials.pdf]

Figure S1. Dynamics of overall publications on flavonoid research extracted from Pubmed timeline metrics. This plot represents information from 1946 to mid-2018

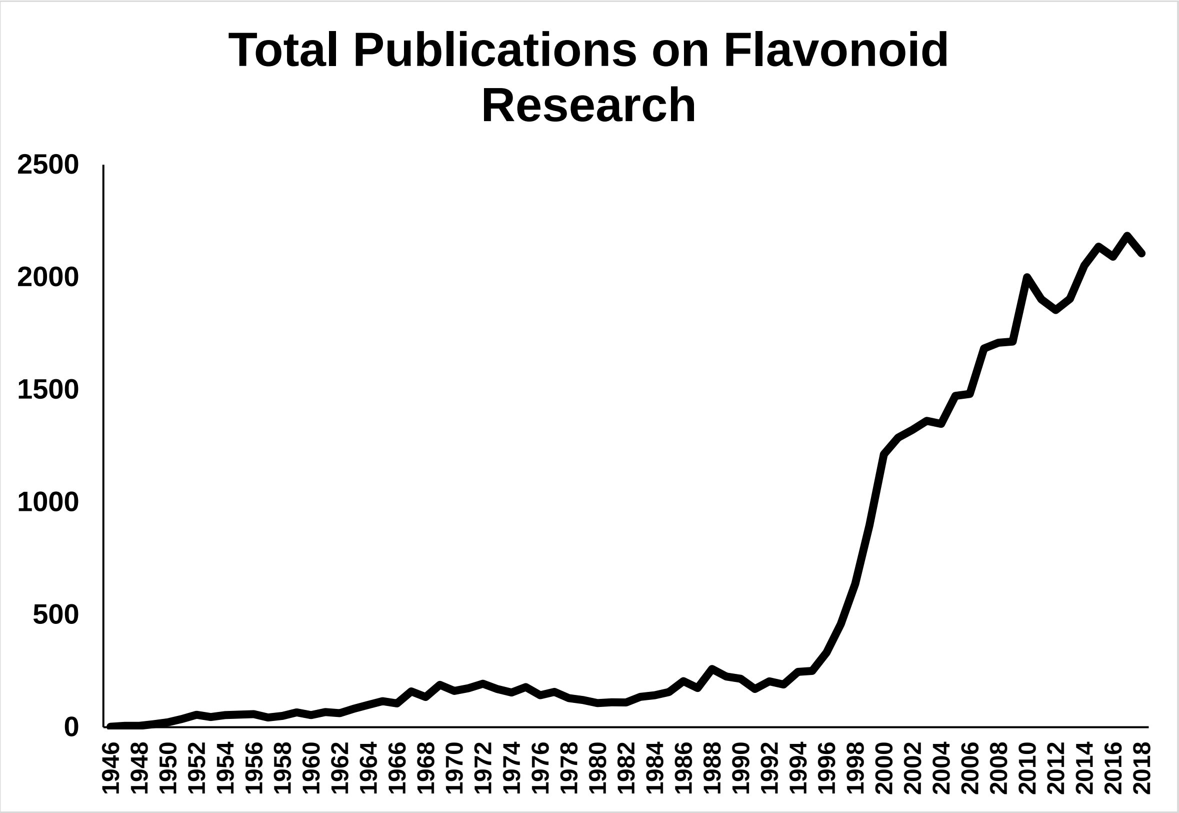

Figure S2. Dynamics of meta-analysis and systematic review-based publications on flavonoid research extracted from Pubmed timeline metrics. This plot represents information from 1990 to mid-2018

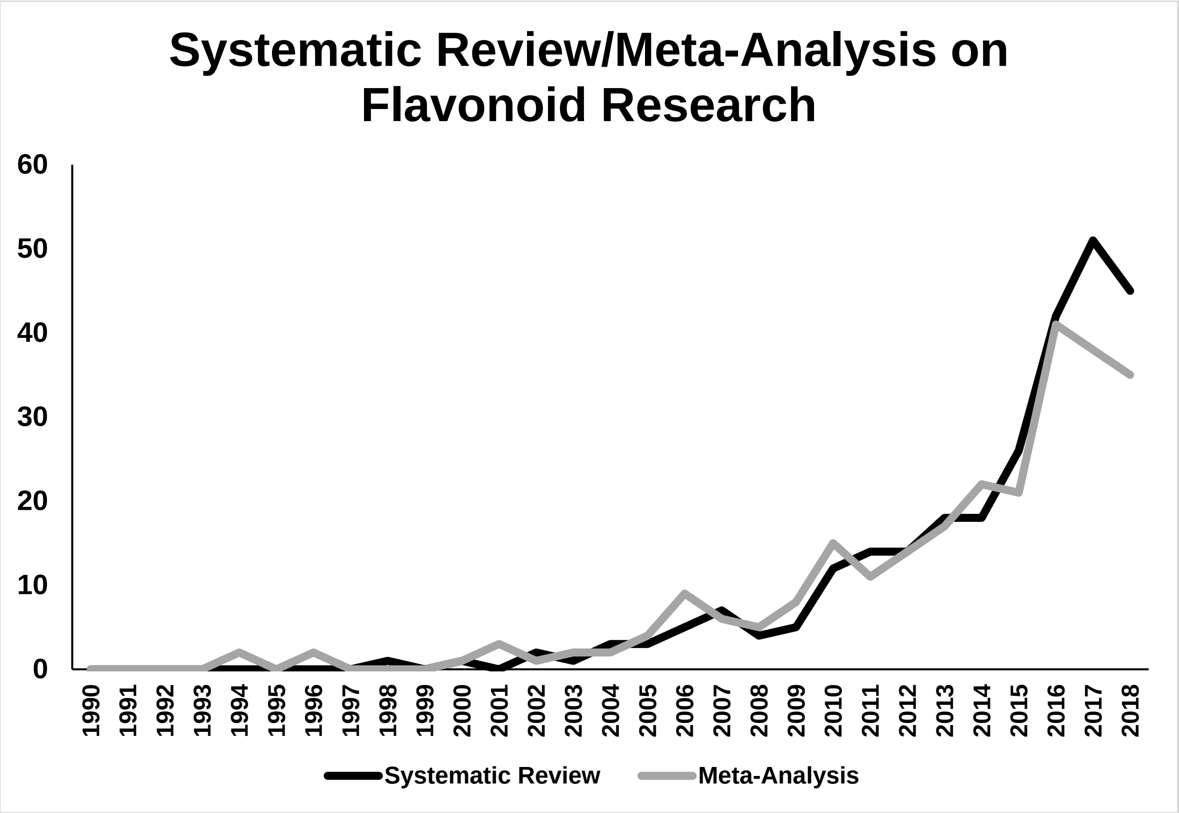

Table S1. Major outcomes reported in selected articles in diabetes animal models

No	Compound name	Model/Dosing	Follow up period	Major outcomes*				Reference
				Blood markers	Gene expression / protein content	Tissue/cell content	Whole-animal experiments	
1	Naringin	Type 2 diabetes<br>(STZ/NA injection)<br>100 mg/kg	4 weeks	-Insulin: ↑<br>-C-peptide: ↑↑<br>-TCh: ↓<br>-TG: ↓<br>-HDL: ↑<br>-LDL: ↓↓<br>-VDLD: NS<br>-FFA: ↓	-Insulin receptor: ↑<br>-GLUT-4: ↑<br>-Adiponectin: ↑	*Tissue content and activity*<br>-Liver glycogen: ↑<br>-Liver G6P: ↓↓<br>-Liver GPh: ↓<br><br>*Tissue oxidative stress and antioxidant*<br>-Liver LPO: ↓<br>-Liver GSH: ↑<br>-Liver GPx: ↑<br>-Liver GST: ↑	*Glucose tolerance test*<br><br>-OGTT: ↓ (glucose peak)	19
	Naringenin			-Insulin: ↑<br>-C-peptide: ↑↑<br>-TCh: ↓<br>-TG: ↓<br>-HDL: ↑<br>-LDL: ↓↓<br>-VDLD: ↓<br>-FFA: ↓	-Insulin receptor: ↑<br>-GLUT-4: ↑<br>-Adiponectin: ↑	*Tissue content and activity*<br>-Liver glycogen: ↑<br>-Liver G6P: ↓↓<br>-Liver GPh: ↓<br><br>*Tissue oxidative stress and antioxidant*<br>-Liver LPO: ↓<br>-Liver GSH: ↑<br>-Liver GPx: ↑<br>-Liver GST: ↑	*Glucose tolerance test*<br><br>-OGTT: ↓↓ (glucose peak)	
2	Hesperidin	Type 2 diabetes<br>(GK rats)<br>10 mg/kg (1%)	4 weeks	-Glucose: ↓↓<br>(after 2 weeks)<br>-Insulin: ↓↓↓<br>-Adiponectin: ↑<br>-TG: ↓↓<br>-TCh: ↓<br>-HDL/TCh ratio: ↑↑	-PPAR-α: ↑<br>-PPAR-γ: ↑<br>-HMG-CoA reductase: ↓<br>-ACAT1: NS<br>-ACAT2: NS<br>-LDL receptor: ↑	*Tissue enzyme activity*<br>-Liver G6P: ↓↓<br>-Liver GK: NS<br><br>*Tissue content*<br>-Liver TG: ↓↓<br>-Liver TCh: ↓↓	Body weight: NS<br>Food intake: NS	20
3	Hesperidin	Type 1 diabetes<br>(STZ injection)<br>10 mg/kg (1%)	4 weeks	-Glucose: ↓↓<br>(1 day prior to dissection)<br>-Insulin: ↑↑↑<br>-Adiponectin: ↑↑<br>-TG: ↓↓<br>-TCh: ↓↓<br>-HDL: ↑↑<br>-LDL+VLDL: ↓↓<br>-HDL/TCh ratio: ↑↑	N/A	*Tissue enzyme activity*<br>-Liver G6P: ↓↓↓<br>-Liver GK: ↓↓<br><br>*Tissue content*<br>-Liver TG: ↓↓<br>-Liver TCh: ↓↓↓<br><br>*Markers of bone turnover*<br>-Osteocalcin: NS	N/A	21
4	Naringenin	HFD<br>30 mg/kg (3%)	4 weeks	-Leptin<br>WT: ↓↓<br>$Fgf21^{-/-}$: ↓↓<br><br>-Adiponectin<br>WT: NS<br>$Fgf21^{-/-}$: SG<br><br>-TNF-α<br>WT: ↓↓↓ CN<br>$Fgf21^{-/-}$: ↓↓	*WAT gene expression*<br><br>-Pgc1a<br>WT: ↓↓<br>$Fgf21^{-/-}$: ↓↓<br>-Cpt1a<br>WT: SG<br>$Fgf21^{-/-}$: SG<br>-Ucp1<br>WT: ↑↑↑<br>$Fgf21^{-/-}$: SG	*Adipocyte diameter*<br>WT: ↓↓<br>$Fgf21^{-/-}$: ↓↓<br><br>*Total number of adipocyte*<br>WT: ↓↓<br>$Fgf21^{-/-}$: ↓<br><br>*Tissue content*<br>-Liver TG: ↓ (ob/ob)	*Visceral adipose tissue volume*<br>WT: ↓↓<br>$Fgf21^{-/-}$: ↓↓<br><br>*Subcutaneous adipose tissue volume*<br>WT: ↓↓<br>$Fgf21^{-/-}$: ↓↓<br><br>*Glucose tolerance test (GTT)*	22

No	Compound name	Model/Dosing	Follow up period	Major outcomes*				Reference
				Blood markers	Gene expression / protein content	Tissue/cell content	Whole-animal experiments	
				-TG<br>WT: SG<br>*Fgf21*$^{-/-}$: ↓↓↓ CN<br><br>-FFA<br>WT: SG<br>*Fgf21*$^{-/-}$: SG<br><br>-Insulin<br>WT: ↓↓<br>*Fgf21*$^{-/-}$: ↓↓<br><br>-Glucose<br>WT: ↓↓<br>*Fgf21*$^{-/-}$: ↓↓<br><br>*Ob/ob mice*<br>-TG: ↓↓↓<br>-Insulin: ↓<br>-Glucose: ↓	-Pnpla2 (Atgl)<br>WT: ↑↑↑ CN<br>*Fgf21*$^{-/-}$: ↑↑↑ CN<br>-Lipe (Hsl)<br>WT: ↑↑↑ CN<br>*Fgf21*$^{-/-}$: ↑↑↑ CN<br>-Ppara<br>WT: SG<br>*Fgf21*$^{-/-}$: ↑↑↑ CN<br>-Chrebp<br>WT: NS<br>*Fgf21*$^{-/-}$: NS<br>-Pparg<br>WT: SG<br>*Fgf21*$^{-/-}$: SG<br>-Lepr<br>WT: NS<br>*Fgf21*$^{-/-}$: NS<br><br>*Liver gene expression*<br><br>-Pgc1a<br>WT: ↑↑<br>*Fgf21*$^{-/-}$: ↑↑<br>-Cpt1a<br>WT: ↑↑<br>*Fgf21*$^{-/-}$: ↑↑<br>-Acox<br>WT: SG<br>*Fgf21*$^{-/-}$: SG<br>-Ppara<br>WT: SG<br>*Fgf21*$^{-/-}$: SG<br>-Srebf1 (Srebp1c)<br>WT: ↓↓<br>*Fgf21*$^{-/-}$: ↓↓<br>-Acc1<br>WT: ↓↓<br>*Fgf21*$^{-/-}$: SG<br>-Acc2<br>WT: NS<br>*Fgf21*$^{-/-}$: SG<br>-Scd1<br>WT: ↓↓<br>*Fgf21*$^{-/-}$: SG<br>-Pparg<br>WT: SG<br>*Fgf21*$^{-/-}$: SG<br>-Lepr<br>WT: ↑↑↑ CN<br>*Fgf21*$^{-/-}$: SG	-Liver TG: ↓↓ (WT)<br>-Liver TG: ↓↓ (*Fgf21*$^{-/-}$)<br><br>-Quadriceps TG: ↓↓ (WT)<br>-Quadriceps TG: ↓↓ (*Fgf21*$^{-/-}$)	WT: ↓↓ (all curve)<br>*Fgf21*$^{-/-}$: ↓↓ (all curve)<br><br>*Insulin tolerance test (ITT)*<br>WT: ↓↓<br>*Fgf21*$^{-/-}$: ↓↓<br><br>*AUC - GTT*<br>WT: NS<br>*Fgf21*$^{-/-}$: ↓↓<br><br><br>*AUC - ITT*<br>WT: NS<br>*Fgf21*$^{-/-}$: ↓↓<br><br>*Glucose tolerance test (ob/ob)*<br>-OGTT: ↓<br>-AUC: ↓<br><br>Body weight: NS (ob/ob)<br>Adipocity index: NS (ob/ob)	
5	Naringenin	30 mg/kg (3%)	12 weeks	-TCh<br>Chow: ↓<br>Chow+Nar: ↓↓	N/A	Aorta lipid content *Ldlr*$^{-/-}$ mice<br><br>-Free cholesterol (FC)	*Glucose tolerance test*<br>Chow: NS<br>Chow+Nar: ↓↓↓	23

<table>
<tr><th rowspan="2">No</th><th rowspan="2">Compound name</th><th rowspan="2">Model/Dosing</th><th rowspan="2">Follow up period</th><th colspan="4">Major outcomes*</th><th rowspan="2">Reference</th></tr>
<tr><th>Blood markers</th><th>Gene expression / protein content</th><th>Tissue/cell content</th><th>Whole-animal experiments</th></tr>
<tr><td></td><td></td><td></td><td></td><td>-TG
Chow: ↓
Chow+Nar: ↓↓

-VLDL-Cholesterol
Chow: ↓
Chow+Nar: ↓↓↓

-LDL-Cholesterol
Chow: ↓
Chow+Nar: ↓↓↓

-VLDL-Triglycerides
Chow: ↓
Chow+Nar: ↓↓↓

-Insulin
Chow: NS
Chow+Nar: ↓↓↓

-Glucose
Chow: NS
Chow+Nar: ↓↓↓

-NEFA
Chow: ↓
Chow+Nar: ↓</td><td></td><td>Chow: ↓
Chow+Nar: ↓↓

- CE
Chow: ↓
Chow+Nar: ↓↓↓

-Triglycerides (TG)
Chow: NS
Chow+Nar: ↓↓↓

-Aorta lesion area (size)
Chow: ↓
Chow+Nar: ↓↓</td><td>Insulin tolerance test
Chow: NS
Chow+Nar: ↓↓↓

HOMA-IR
Chow: NS
Chow+Nar: ↓↓↓</td><td></td></tr>
<tr><td>6</td><td>Hesperetin</td><td>40 mg/kg</td><td>6 weeks</td><td>Plasma content
-Glucose: ↓
-Insulin: ↑

Enzyme activity
-SOD: ↑
-CAT: ↑
-GST: ↑
-GPx: ↑</td><td>N/A</td><td>Glucose metabolic enzymes (activity)
-Liver GK: ↑
-Liver G6PD: ↑
-Liver G6P: ↓
-Liver FBP: ↓
-Liver glycogen: ↑

Antioxidant enzymes (activity)
-Pancreas SOD: ↑
-Pancreas CAT: ↑
-Pancreas GST: ↑
-Pancreas GPx: ↑

Liver lipid profile (content)
-TCh: ↓
-TG: ↓
-FFA: ↓
-Phospholipids: ↓

Liver toxicity markers (activity)
-AST: ↓
-ALT: ↓</td><td>N/A</td><td>17</td></tr>
</table>

No	Compound name	Model/Dosing	Follow up period	Major outcomes*				Reference
				Blood markers	Gene expression / protein content	Tissue/cell content	Whole-animal experiments	
						-ALP: ↓<br><br>Kidney function markers<br>-Urea: ↓<br>-Creatinine: ↓<br>-Uric acid: ↓<br><br>*Pancreas staining area*<br>-Insulin positive: ↑		
7	Neohesperidin	50 mg/kg	6 weeks	*Plasma content*<br>-Fasting glucose: ↓↓ (after 5 weeks)<br>-Fasting glucose: ↓ (after 6 weeks)<br><br>*Biochemical parameters at the end (6 weeks)*<br>-Glucose: ↓↓<br>-TCh: ↓<br>-TG: ↓↓<br>-ALT: ↓<br>-AST: NS<br>-GSP: ↓↓↓<br>-HbA1c: NS<br>-Leptin: ↓↓<br>-Adiponectin: SG	*Liver genes associated with PPAR and AMPK pathways*<br>-PPAR-α: NS<br>-PPAR-γ: NS<br>-SCD-1: ↓↓<br>-CPT-1: NS<br>-AP-2: NS<br>-UCP-2: NS<br>-SREBP-1c: NS<br>-ACC-α: NS<br>-FAS: ↓↓↓<br>-ACOX: ↑↑↑<br>-ACS: NS<br>-PGC-1α: NS<br><br>*Liver phosphorylated proteins*<br>-p-AMPK/AMPK: ↑↑↑	*Liver content*<br>-Glycogen: NS<br>-TG: ↓<br>-TCh: NS<br><br>*Adipocyte parameters*<br>-Relative adipocyte size: ↓	*Glucose and insulin tolerance test*<br>-OGTT: ↓ (all time points)<br>-ITT: ↓ (all time points)<br><br>*Insulin resistance index*<br>HOMA-IR: ↓↓	18
8	Hesperidin	0.2 g/kg	5 weeks	*Plasma content*<br>-Insulin: ↑<br>-C-peptide: ↑<br>-Leptin: ↑	N/A	*Liver enzyme activity*<br>-GK: ↑<br>-PEPC: ↓<br>-G6P: ↓<br><br>*Liver content*<br>-Glycogen: ↑	*Blood markers through time (curve)*<br>-Glucose: ↓↓ ↓ (after 3rd week)	24
	Naringin			*Plasma content*<br>-Insulin: ↑<br>-C-peptide: ↑<br>-Leptin: ↑	N/A	*Liver enzyme activity*<br>-GK: ↑<br>-PEPC: ↓<br>-G6P: ↓<br><br>*Liver content*<br>-Glycogen: ↑	*Blood markers through time (curve)*<br>-Glucose: ↓↓↓ (after 3rd week)	
9	Hesperidin	0.2 g/kg	5 weeks	*Plasma content*<br>-Glucose: ↓<br>-FFA: ↓<br>-TG: ↓<br>-TCh: ↓<br>-HDL: NS<br>-HDL/TCh ratio: ↑<br>-Atherogenic index: ↓<br>-PON: ↑	*Liver protein content*<br>-FAS: ↓<br>-G6PD: ↓<br>-PAP: ↓<br>-CPT: ↓<br>-β-oxidation: ↓<br>-HMG-CoA: ↓<br>-ACAT: ↓<br>-GLUT-2: ↓↓<br>-PPAR-γ: ↑↑↑	*Liver content*<br>-TG: ↓<br>-TCh: ↓<br><br>*Fecal content*<br>-TG: ↑<br>-TCh: ↑	N/A	25

No	Compound name	Model/Dosing	Follow up period	Major outcomes*				Reference
				Blood markers	Gene expression / protein content	Tissue/cell content	Whole-animal experiments	
					Adipocyte protein content<br>-GLUT-4: ↑↑<br>-PPAR-γ: ↑↑↑<br><br>*Liver gene expression (mRNA)*<br>-GK: ↑↑↑<br>-G6P: NS<br>-PEPCK: NS			
	Naringin			*Plasma content*<br>-Glucose: ↓<br>-FFA: ↓<br>-TG: ↓<br>-TCh: ↓<br>-HDL: NS<br>-HDL/TCh ratio: ↑<br>-Atherogenic index: ↓<br>-PON: ↑	*Liver protein content*<br>-FAS: ↓<br>-G6PD: ↓<br>-PAP: ↓<br>-CPT: ↓<br>-β-oxidation: ↓<br>-HMG-CoA: ↓<br>-ACAT: ↓<br>-GLUT-2: ↓↓<br>-PPAR-γ: ↑↑↑<br><br>*Adipocyte protein content*<br>-GLUT-4: ↑↑<br>-PPAR-γ: ↑↑↑<br><br>*Live gene expression (mRNA)*<br>-GK: ↑↑↑<br>-G6P: ↓<br>-PEPCK: ↓	*Liver content*<br>-TG: ↓<br>-TCh: ↓<br><br>*Fecal content*<br>-TG: ↑<br>-TCh: ↑	N/A	
10	Hesperidin	50 mg/kg	4 weeks	*Plasma content*<br>-Insulin: ↑<br>-HbAc1: ↓	*Adipocyte gene expression*<br>-GLUT-4: ↑↑<br><br>*Adipocyte protein content*<br>-GLUT-4: ↑↑	*Liver content*<br>-Glycogen: ↑<br><br>*Muscle content*<br>- Glycogen: ↑<br><br>*Concentration-dependent glucose uptake in diaphragm*<br>-0.2 mg/ml (-insulin): ↑<br>-0.2 mg/ml (+insulin): NS<br>-1.0 mg/ml (-insulin): ↑<br>-1.0 mg/ml (+insulin): ↑<br><br>*Concentration-dependent intestinal glucose absorption*<br>-5 mg/ml: NS<br>-10 mg/ml: ↓<br>-20 mg/ml: ↓<br><br>*Liver enzyme activity*<br>-G6P: ↓<br>-GPh: ↓↓<br>-FBP: ↓<br>-GK: ↑	*Glucose tolerance test*<br>-OGGT: ↓↓ (all time points)<br><br>*Insulin resistance index*<br>-HOMA-IR: ↓	26

No	Compound name	Model/Dosing	Follow up period	Major outcomes*				Reference
				Blood markers	Gene expression / protein content	Tissue/cell content	Whole-animal experiments	
						Concentration-dependent insulin release from pancreatic islets<br>-Glucose 4 mM: ↑↑ (all concentrations)<br>-Glucose 20 mM: ↑ (all concentrations)<br><br>*Concentration-dependent nitric oxide production in pancreatic islets*<br>-Glucose 20 mM+IL-1β: ↓ (0.5-2.0 mg)		
	Naringin			*Plasma content*<br>-Insulin: ↑<br>-HbAc1: ↓	*Adipocyte gene expression*<br>-GLUT-4: ↑↑<br><br>*Adipocyte protein content*<br>-GLUT-4: ↑↑	*Liver content*<br>-Glycogen: ↑<br><br>*Muscle content*<br>- Glycogen: ↑<br><br>*Concentration-dependent glucose uptake in diaphragm*<br>-0.2 mg/ml (-insulin): ↑<br>-0.2 mg/ml (+insulin): NS<br>-1.0 mg/ml (-insulin): ↑<br>-1.0 mg/ml (+insulin): ↑<br><br>*Concentration-dependent intestinal glucose absorption*<br>-5 mg/ml: NS<br>-10 mg/ml: ↓<br>-20 mg/ml: ↓<br><br>*Liver enzyme activity*<br>-G6P: ↓<br>-GPh: ↓<br>-FBP: ↓<br>-GK: ↑<br><br>*Concentration-dependent insulin release from pancreatic islets*<br>-Glucose 4 mM: ↑↑ (all concentrations)<br>-Glucose 20 mM: ↑ (all concentrations)<br><br>*Concentration-dependent nitric oxide production in pancreatic islets*<br>-Glucose 20 mM+IL-1β: ↓ (0.5-2.0 mg)	*Glucose tolerance test*<br>-OGGT: ↓↓ (all time points)<br><br>*Insulin resistance index*<br>-HOMA-IR: ↓	

No	Compound name	Model/Dosing	Follow up period	Major outcomes*				Reference
				Blood markers	Gene expression / protein content	Tissue/cell content	Whole-animal experiments	
11	Naringin	100 mg/kg	4 weeks	*Plasma content*<br>-Glucose: NS<br>-TCh: ↓↓<br>-TG: ↓↓<br>-HDL: SG<br>-LDL: ↓↓	*Protein content*<br>-eNOS: ↑↑<br>-p-eNOS/eNOS ratio: ↑<br>-Nitrotyrosine: ↓↓↓	*Vascular reactivity of pre-treated animals*<br>-ACh: ↑↑ (+endothelium)<br>-SNP: SG (-endothelium)<br><br>*Vascular relaxation after eNOS and COX inhibitors*<br>-NG-nitro-L-arginine: NS<br>-Indomethacin: ↑↑	Body weight: NS	27
12	Naringenin	10, 30 mg/kg<br>(1% or 3%)	4 weeks	*Plasma content*<br><br>-TCh<br>Naringenin 1%: ↓<br>Naringenin 3%: ↓<br><br>-TG<br>Naringenin 1%: ↓<br>Naringenin 3%: ↓↓<br><br>*Protein liquid chromatography analysis*<br><br>-VLDL-Ch:<br>Naringenin 1%: NS<br>Naringenin 3%: ↓↓<br><br>-LDL-Ch fraction (at peak):<br>Naringenin 1%: NS<br>Naringenin 3%: ↓<br><br>-HDL-Ch<br>Naringenin 1%: NS<br>Naringenin 3%: NS<br><br>-VLDL-TG:<br>Naringenin 1%: NS<br>Naringenin 3%: ↓<br><br>-NEFA<br>Naringenin 1%: SG<br>Naringenin 3%: SG<br><br>*Secretion after chemical induction (naringenin 3%)*<br>-TG: ↓↓<br>-ApoB100: ↓↓<br><br>*Lipase activity*<br>-LPL: ↑↑↑ CN	*Liver gene expression*<br><br>-Srebp1c<br>Naringenin 1%: ↓<br>Naringenin 3%: ↓↓<br><br>-Pgc1α<br>Naringenin 1%: NS<br>Naringenin 3%: ↓↓<br><br>-Pparα<br>Naringenin 1%: SG<br>Naringenin 3%: SG<br><br>-Cpt1α<br>Naringenin 1%: ↑↑<br>Naringenin 3%: ↑↑<br><br>-Aco<br>Naringenin 1%: ↑↑ CN<br>Naringenin 3%: ↑↑ CN<br><br>-Mitochondrial DNA<br>Naringenin 1%: ↑↑ CN<br>Naringenin 3%: ↑↑ CN<br><br>*Muscle gene expression*<br><br>-Pgc1α<br>Naringenin 1%: SG<br>Naringenin 3%: SG<br><br>-Cpt1β<br>Naringenin 1%: SG<br>Naringenin 3%: SG<br><br>-Ucp1<br>Naringenin 1%: SG<br>Naringenin 3%: SG<br><br>-Ucp3<br>Naringenin 1%: ↓↓↓ CN<br>Naringenin 3%: ↓↓↓ CN<br><br>-Srebp1c	*Intestinal TG mass*<br>Naringenin 1%: ↓<br>Naringenin 3%: ↓↓<br><br>*Liver content*<br><br>-TCh<br>Naringenin 1%: ↓<br>Naringenin 3%: ↓<br><br>-Free Ch<br>Naringenin 1%: SG<br>Naringenin 3%: SG<br><br>-CE<br>Naringenin 1%: ↓<br>Naringenin 3%: ↓<br><br>-TG mass<br>Naringenin 1%: NS<br>Naringenin 3%: ↓↓<br><br>-FA synthesis<br>Naringenin 1%: ↓<br>Naringenin 3%: ↓<br><br>-TG synthesis<br>Naringenin 1%: ↓<br>Naringenin 3%: ↓<br><br>-Ch synthesis<br>Naringenin 1%: NS<br>Naringenin 3%: ↓↓<br><br>-CE synthesis<br>Naringenin 1%: ↓↓<br>Naringenin 3%: ↓↓<br><br>-FA oxidation<br>Naringenin 1%: ↑↑<br>Naringenin 3%: ↑↑<br><br>*Pancreas content*	*Body weight changes*<br>Naringenin 1%: NS<br>Naringenin 3%: ↓↓<br><br>*Caloric intake*<br>Naringenin 1%: SG<br>Naringenin 3%: SG<br><br>*Intestinal TG absorption*<br>Naringenin 1%: SG<br>Naringenin 3%: SG<br><br>*Intestinal TCh absorption*<br>Naringenin 1%: SG<br>Naringenin 3%: SG<br><br>*Insulin resistance index*<br><br>-HOMA-IR<br>Naringenin 1%: ↓↓<br>Naringenin 3%: ↓↓<br><br>*Glucose and insulin tolerance test*<br><br>-OGTT<br>Naringenin 3%: ↓↓↓<br>(at peak)<br><br>-ITT<br>Naringenin 3%: ↓↓<br>(all time points)<br><br>-ITT - AUC<br>Naringenin 3%: ↓↓<br><br>-ITT (corrected for fasting glucose)<br>Naringenin 3%: ↓ (trend at peak)<br><br>*Animal body metrics*<br>-Total adipose tissue<br>Naringenin 1%: ↓	18

No	Compound name	Model/Dosing	Follow up period	Major outcomes*				Reference
				Blood markers	Gene expression / protein content	Tissue/cell content	Whole-animal experiments	
					Naringenin 1%: NS<br>Naringenin 3%: ↓↓	-Insulin<br>Naringenin 1%: ↓<br>Naringenin 3%: ↓↓<br><br>-Glucose<br>Naringenin 1%: ↓<br>Naringenin 3%: ↓↓<br><br>*Pancreas islet size*<br>Naringenin 3%: ↓↓<br><br>*Muscle content*<br><br>-TG mass<br>Naringenin 1%: ↓<br>Naringenin 3%: ↓↓<br><br>-CE mass<br>Naringenin 1%: ↓<br>Naringenin 3%: ↓<br><br>-FA oxidation<br>Naringenin 1%: SG<br>Naringenin 3%: SG<br><br>-FA synthesis<br>Naringenin 1%: ↓↓<br>Naringenin 3%: ↓↓<br><br>*Deoxyglucose uptake*<br>Naringenin 1%: NS<br>Naringenin 3%: ↑<br><br>*Adipose tissue histological metrics*<br><br>-Adipocyte diameter (naringenin 3%)<br>1-30 µm: ↑↑↑ CN<br>31-50 µm: ↑<br>51-70 µm: ↑↑<br>71-90 µm: ↓<br>91-120 µm: ↓<br><br>*Adipose tissue content*<br><br>-Leptin<br>Naringenin 1%: ↓<br>Naringenin 3%: ↓↓<br><br>*Adipose tissue calorimetric parameters*	Naringenin 3%: ↓↓<br><br>-Visceral adipose tissue<br>Naringenin 1%: ↓<br>Naringenin 3%: ↓↓	

No	Compound name	Model/Dosing	Follow up period	Major outcomes*				Reference
				Blood markers	Gene expression / protein content	Tissue/cell content	Whole-animal experiments	
						-Energy expenditure<br>Naringenin 3%: ↑↑↑ (from 600 min to the end)<br><br>-Energy expenditure - AUC<br>Naringenin 3%: ↑↑↑		
13	Nobiletin	10, 30 mg/kg<br>(0.1% or 0.3%)	8-26 weeks	*Plasma content ($Ldlr^{-/-}$ mice fed Western diet 8 weeks)*<br><br>-TG<br>Nobiletin 0.1%: ↓<br>Nobiletin 0.3%: ↓<br><br>-Chol<br>Nobiletin 0.1%: ↓<br>Nobiletin 0.3%: ↓<br><br>-NEFA<br>Nobiletin 0.1%: ↓<br>Nobiletin 0.3%: ↓↓<br><br>-Glycerol<br>Nobiletin 0.1%: ↓<br>Nobiletin 0.3%: ↓↓<br><br>-TG secretion<br>Nobiletin 0.3%: ↓↓<br><br>-TG secretion rate<br>Nobiletin 0.3%: ↓↓↓<br><br>-Ketones<br>Nobiletin 0.3%: ↑↑<br><br>-Insulin<br>Nobiletin 0.1%: NS<br>Nobiletin 0.3%: ↓↓<br><br>-Glucose<br>Nobiletin 0.1%: ↓<br>Nobiletin 0.3%: ↓↓<br><br>-Leptin<br>Nobiletin 0.1%: NS<br>Nobiletin 0.3%: ↓↓<br><br>*Plasma content ($Ldlr^{-/-}$ mice fed Western diet 26 weeks)*<br><br>-TG	*Phosphorylated ERK protein content*<br><br>-ERK phosphorylation<br>Nobiletin 2.5 μM: ↑<br>Nobiletin 5 μM: ↑↑<br>Nobiletin 10 μM: ↑↑<br>Nobiletin 20 μM: ↑↑<br><br>*Gene expression (HepG2)*<br><br>-MTP<br>Nobiletin 10 μM + UO126 (active): ↑↑<br>Nobiletin 10 μM + UO124 (null): NS<br><br>-LDLR<br>Nobiletin 10 μM + UO126 (active): ↓↓<br>Nobiletin 10 μM + UO124 (null): NS<br><br>-<br>Nobiletin 10 μM + UO126 (active): ↑↑<br>Nobiletin 10 μM + UO124 (null): ↑<br><br>-DGAT2<br>Nobiletin 10 μM + UO126 (active): ↑<br>Nobiletin 10 μM + UO124 (null): NS<br><br>*Lipid synthesis and content (HepG2 cells)*<br><br>-CPT1α<br>Nobiletin 10 μM: ↑↑<br><br>-ACOX<br>Nobiletin 10 μM: NS<br><br>-PGC1α<br>Nobiletin 10 μM: ↑↑	*Lipoprotein secretion (HepG2 cells)*<br><br>- ApoB100<br>Nobiletin 1 μM: NS<br>Nobiletin 2.5 μM: NS<br>Nobiletin 5 μM: ↓↓<br>Nobiletin 10 μM: ↓↓<br>Nobiletin 20 μM: ↓↓<br><br>*Lipoprotein secretion – mechanistic experiments (HepG2 cells)*<br><br>- UO126 (MEK 1/2 inhibitor)<br>Nobiletin 10 μM + UO126 (active): ↑↑<br>Nobiletin 10 μM + UO124 (null): NS<br><br>- SB203580 (p38 MAPK inhibitor)<br>Nobiletin 10 μM + SB203580 (active): ↑↑<br>Nobiletin 10 μM + SB248080 (null): NS<br><br>-ERK phosphorylation (peak)<br>Nobiletin 10 μM: ↑↑<br><br>*Lipid synthesis and content (HepG2 cells)*<br><br>-LDL uptake<br>Nobiletin 5 μM: ↓↓<br>Nobiletin 10 μM: ↓<br><br>-MTP activity<br>Nobiletin 10 μM: ↓↓<br><br>-FA synthesis<br>Nobiletin 10 μM: ↓↓<br><br>-TG synthesis<br>Nobiletin 10 μM: ↓↓	*Glucose and insulin tolerance test*<br><br>-OGTT - AUC<br>Nobiletin 0.3%: ↓↓<br><br>-ITT - AUC<br>Nobiletin 0.3%: ↓↓<br><br>-Glucose infusion rate - clamp<br>Nobiletin 0.3%: ↑↑<br><br>-Whole-body glucose uptake - clamp<br>Nobiletin 0.3%: ↑↑<br><br>-Hepatic glucose production - clamp<br>Nobiletin 0.3%: ↓↓<br><br>-%Hepatic glucose production - clamp<br>Nobiletin 0.3%: ↑↑<br><br>-Pyruvate tolerance test<br>Nobiletin 0.3%: ↓↓ (at 120 min)<br><br>*Energy expenditure*<br><br>-Total energy expenditure (24 h)<br>Nobiletin 0.3%: ↑↑ CN<br><br>-Energy expenditure (Dark cycle)<br>Nobiletin 0.3%: ↑↑↑ CN	28

No	Compound name	Model/Dosing	Follow up period	Major outcomes*				Reference
				Blood markers	Gene expression / protein content	Tissue/cell content	Whole-animal experiments	
				Nobiletin 0.3%: ↓↓<br><br>-Chol<br>Nobiletin 0.3%: ↓↓<br><br>-Insulin<br>Nobiletin 0.3%: ↓↓<br><br>-Glucose<br>Nobiletin 0.3%: ↓↓	*Liver lipid metabolism genes*<br><br>-Srebf1c<br>Nobiletin 0.1%: ↓<br>Nobiletin 0.3%: ↓↓<br><br>-Mttp<br>Nobiletin 0.3%: NS<br><br>-Dgat1<br>Nobiletin 0.3%: NS<br><br>-Dgat2<br>Nobiletin 0.3%: SG<br><br>-Cpt1α<br>Nobiletin 0.3%: ↑↑↑<br><br>-Pgc1α<br>Nobiletin 0.3%: ↑↑	-TG mass<br>Nobiletin 10 μM: ↓↓<br><br>-Palmitate oxidized<br>Nobiletin 10 μM: NS<br><br>*Liver content*<br>*(Ldlr$^{-/-}$ mice fed Western diet 8 weeks)*<br><br>-TG mass<br>Nobiletin 0.1%: ↓<br>Nobiletin 0.3%: ↓↓<br><br>-CE mass<br>Nobiletin 0.1%: ↓↓<br>Nobiletin 0.3%: ↓↓<br><br>*Lipid content in intestine*<br><br>-TG mass<br>Nobiletin 0.1%: ↓↓<br>Nobiletin 0.3%: ↓↓<br><br>Liver β-oxidation<br>Nobiletin 0.3%: ↑↑↑<br><br>*Lipid content in isolated tissue*<br><br>-TG content – gastrocnemius muscle<br>Nobiletin 0.1%: ↓↓<br>Nobiletin 0.3%: ↓↓<br><br>-TG content – Quadriceps muscle<br>Nobiletin 0.1%: ↓↓<br>Nobiletin 0.3%: ↓↓<br><br>*Adiposity parameters*<br><br>-Adiposity index<br>Nobiletin 0.1%: NS<br>Nobiletin 0.3%: ↓↓<br><br>-Mean adipocyte diameter<br>Nobiletin 0.1%: ↓<br>Nobiletin 0.3%: ↓<br><br>*Liver content*<br>*(Ldlr$^{-/-}$ mice fed Western diet 26 weeks)*		

No	Compound name	Model/Dosing	Follow up period	Major outcomes*				Reference
				Blood markers	Gene expression / protein content	Tissue/cell content	Whole-animal experiments	
						-TG mass<br>Nobiletin 0.3%: ↓↓<br><br>-CE mass<br>Nobiletin 0.3%: ↓↓<br><br>*Modifications in aortic sinus (Ldlr$^{-/-}$ mice fed Western diet 26 weeks)*<br><br>-Lesion area (X10^5)<br>Nobiletin 0.3%: ↓		
14	Naringenin	6, 12.5, 25 mg/kg	6 weeks	*Plasma content (at 45 days)*<br><br>-Glucose: ↓<br>-Insulin: ↓<br>-ALT: ↓<br>-AST: ↓<br>-ALP: ↓<br>-TG: ↓<br>-FFA: ↓<br>-TCh: ↓<br>-LDL: ↓<br>-VLDL: ↓<br>-HDL: ↓	*Gene expression in adipose tissue*<br><br>-GLUT-4<br>Naringenin 25 mg/kg: ↑<br><br>-TNF-α<br>Naringenin 25 mg/kg: ↓<br><br>*Gene expression in skeletal muscle*<br><br>-GLUT-4<br>Naringenin 25 mg/kg: ↑<br><br>*Protein content in adipose tissue*<br><br>-GLUT-4<br>Naringenin 25 mg/kg: ↑<br><br>-TNF-α<br>Naringenin 25 mg/kg: ↓<br><br>*Gene expression in skeletal muscle*<br><br>-GLUT-4<br>Naringenin 25 mg/kg: ↑	*Lipid peroxidation – TBARS*<br><br>-Plasma: ↓<br>-Liver: ↓<br>-Pancreas: ↓<br><br>*Lipid peroxidation – Hydroperoxide*<br><br>-Plasma: ↓<br>-Liver: ↓<br>-Pancreas: ↓<br><br>*Antioxidant enzymes content – Liver*<br><br>-SOD: ↑<br>-Catalase: ↑<br>-GPx: ↑<br><br>*Antioxidant enzymes content – Pancreas*<br><br>-SOD: ↑<br>-Catalase: ↑<br>-GPx: ↑	*Whole-body biometric parameters*<br><br>-Body weight<br>Naringenin 25 mg/kg: ↓	29
15	Naringin	50, 100, 200 mg/kg	3 weeks	*Plasma content*<br><br>-Glucose<br>Naringin 50 mg/kg: ↓<br>Naringin 100 mg/kg: ↓<br>Naringin 200 mg/kg: ↓<br><br>-Insulin<br>Naringin 50 mg/kg: ↓<br>Naringin 100 mg/kg: ↓	*Enzymatic activity in liver*<br><br>-CPT<br>Naringin 50 mg/kg: ↑↑<br>Naringin 100 mg/kg: ↑↑↑ CN<br>Naringin 200 mg/kg: ↑↑↑ CN<br><br>*Gene expression in liver*	*Kidney content*<br><br>-ACE<br>Naringin 50 mg/kg: ↓↓↓<br>Naringin 100 mg/kg: ↓↓↓<br>Naringin 200 mg/kg: ↓↓↓<br><br>*PON activity*<br><br>-Plasma	N/A	30

No	Compound name	Model/Dosing	Follow up period	Major outcomes*				Reference
				Blood markers	Gene expression / protein content	Tissue/cell content	Whole-animal experiments	
				Naringin 200 mg/kg: ↓<br><br>-Bicarbonate<br>Naringin 50 mg/kg: ↓<br>Naringin 100 mg/kg: ↓<br>Naringin 200 mg/kg: ↓<br><br>-FFA<br>Naringin 50 mg/kg: ↓<br>Naringin 100 mg/kg: ↓<br>Naringin 200 mg/kg: ↓<br><br>-Amylase<br>Naringin 50 mg/kg: ↓<br>Naringin 100 mg/kg: ↓<br>Naringin 200 mg/kg: ↓<br><br>-DPP IV<br>Naringin 50 mg/kg: ↓↓<br>Naringin 100 mg/kg: ↓↓<br>Naringin 200 mg/kg: ↓↓<br><br>-α-HBD<br>Naringin 50 mg/kg: ↓<br>Naringin 100 mg/kg: ↓<br>Naringin 200 mg/kg: ↓<br><br>-TC<br>Naringin 50 mg/kg: ↓<br>Naringin 100 mg/kg: ↓<br>Naringin 200 mg/kg: ↓<br><br>-TG<br>Naringin 50 mg/kg: ↓<br>Naringin 100 mg/kg: ↓<br>Naringin 200 mg/kg: ↓<br><br>-HDL-C<br>Naringin 50 mg/kg: ↓<br>Naringin 100 mg/kg: ↓<br>Naringin 200 mg/kg: ↓	-Hmgcr<br>Naringin 50 mg/kg: NS<br>Naringin 100 mg/kg: NS<br>Naringin 200 mg/kg: NS<br><br>-Scarb1<br>Naringin 50 mg/kg: ↑<br>Naringin 100 mg/kg: ↑↑<br>Naringin 200 mg/kg: ↑↑<br><br>-Ahr<br>Naringin 50 mg/kg: ↑<br>Naringin 100 mg/kg: ↑<br>Naringin 200 mg/kg: ↑↑<br><br>-Lipc<br>Naringin 50 mg/kg: ↑<br>Naringin 100 mg/kg: ↑<br>Naringin 200 mg/kg: ↑<br><br>-Lcat<br>Naringin 50 mg/kg: ↑<br>Naringin 100 mg/kg: ↑<br>Naringin 200 mg/kg: ↑	Naringin 50 mg/kg: ↑<br>Naringin 100 mg/kg: ↑<br>Naringin 200 mg/kg: ↑<br><br>-Liver<br>Naringin 50 mg/kg: ↑↑<br>Naringin 100 mg/kg: ↑↑<br>Naringin 200 mg/kg: ↑↑<br><br>*PON activity in lipids*<br><br>-HDL<br>Naringin 50 mg/kg: NS<br>Naringin 100 mg/kg: NS<br>Naringin 200 mg/kg: ↑<br><br>-HDL_3<br>Naringin 50 mg/kg: SG<br>Naringin 100 mg/kg: SG<br>Naringin 200 mg/kg: SG<br><br>-VLDL<br>Naringin 50 mg/kg: ↓<br>Naringin 100 mg/kg: ↓<br>Naringin 200 mg/kg: ↓↓<br><br>-$VLDL_3$<br>Naringin 50 mg/kg: NS<br>Naringin 100 mg/kg: ↓↓<br>Naringin 200 mg/kg: ↓		
16	Diosmin	100 mg/kg	6 weeks	-Insulin: ↑<br>-Glucose: ↓<br><br>*Antioxidant enzymatic activities:*<br>-TARS: ↓<br>-HP: ↓<br>-GSH: ↑<br>-GSSG: ↓	N/A	*Antioxidant enzymatic activities:*<br><br>-Liver<br>TBARS: ↓<br>HP: ↓<br>SOD: ↑<br>GPx: ↑<br>CAT: ↑<br>GST: ↑	N/A	31

No	Compound name	Model/Dosing	Follow up period	Major outcomes*				Reference
				Blood markers	Gene expression / protein content	Tissue/cell content	Whole-animal experiments	
				-GSH/GSSG ratio: ↑<br><br>*Non-enzymatic antioxidant:*<br>Vitamin C: ↑<br>Vitamin E: ↑<br>GSH: ↑		GR: ↑<br><br>-Kidney<br>TBARS: ↓<br>HP: ↓<br>SOD: ↑<br>GPX: ↑<br>CAT: ↑<br>GST: ↑<br>GR: ↑<br><br>*Non-enzymatic antioxidant:*<br><br>-Kidney:<br>Vitamin C: ↑<br>Vitamin E: ↑<br>GSH: ↑<br><br>-Liver:<br>Vitamin C: ↑<br>Vitamin E: ↑<br>GSH: ↑		
17	Diosmin	100 mg/kg	6 weeks	Glucose: ↓<br>HbA1c: ↓<br>CRP: ↓<br><br>*Lipids*<br><br>-Cholesterol: ↓<br>-FF: ↓<br>-TG: ↓<br>-Phospholipids: ↓<br><br>*Lipoproteins*<br><br>-HDL: ↑<br>-LDL: ↓<br>-VLDL: ↓<br><br>*Enzyme activity*<br>-LPL: ↑<br>-LCAT: ↑	N/A	*Liver Lipids*<br><br>-Cholesterol: ↓<br>-FF: ↓<br>-TG: ↓<br>-Phospholipids: ↓<br><br>*Kidney Lipids*<br><br>-Cholesterol: ↓<br>-FF: ↓<br>-TG: ↓<br>-Phospholipids: ↓<br><br>*HMG-CoA/mevalonate ratio*<br><br>-Liver: ↑<br>-Kidney: ↑	N/A	32
18	Targeretin	25, 50, 100 mg/kg	4 weeks	-Glucose<br>Tangeretin 25 mg/kg: ↓<br>Tangeretin 50 mg/kg: ↓<br>Tangeretin 100 mg/kg: ↓<br><br>-Insulin<br>Tangeretin 25 mg/kg: ↑<br>Tangeretin 50 mg/kg: ↑	N/A	*Liver carbohydrate metabolizing enzymes*<br><br>-Hexokinase<br>Tangeretin 100 mg/kg: ↑<br>-Lactate dehydrogenase<br>Tangeretin 100 mg/kg: ↓	Body weight gain: ↑<br>Fluid intake after: ↓<br>*Glucose tolerance test*<br>-OGGT: ↓↓ (all time points)	33

No	Compound name	Model/Dosing	Follow up period	Major outcomes*				Reference
				Blood markers	Gene expression / protein content	Tissue/cell content	Whole-animal experiments	
				Tangeretin 100 mg/kg: ↑<br><br>-Hb<br>Tangeretin 100 mg/kg: ↑<br><br>-Hb1AC<br>Tangeretin 100 mg/kg; ↓		-Pyruvate kinase<br>Tangeretin 100 mg/kg: ↑<br><br>-G6P<br>Tangeretin 100 mg/kg: ↓<br><br>-FBP<br>Tangeretin 100 mg/kg: ↓<br><br>-G6PD<br>Tangeretin 100 mg/kg: ↑<br><br>*Liver Glycogen and glycogen enzyme content:*<br><br>-Glycogen<br>Tangeretin 100 mg/kg: ↑<br><br>-Glycogen synthase<br>Tangeretin 100 mg/kg: ↑<br><br>-Glycogen phosphorylase<br>Tangeretin 100 mg/kg: ↓		
19	Hesperidin	25, 50, 100 mg/kg	4 weeks	-Glucose<br>Hesperidin 25 mg/kg: ↓<br>Hesperidin 50 mg/kg: ↓<br>Hesperidin 100 mg/kg: ↓<br><br>-Insulin<br>Hesperidin 25 mg/kg: ↑<br>Hesperidin 50 mg/kg: ↑<br>Hesperidin 100 mg/kg: ↑<br><br>-Hba1c<br>Hesperidin 25 mg/kg: ↓<br>Hesperidin 50 mg/kg: ↓<br>Hesperidin 100 mg/kg: ↓<br><br>-Hb<br>Hesperidin 25 mg/kg: ↑<br>Hesperidin 50 mg/kg: ↑<br>Hesperidin 100 mg/kg: ↑	N/A	*Liver carbohydrate metabolizing enzymes*<br>Hesperidin 100 mg/kg<br><br>-Hexokinase<br>Hesperidin 100 mg/kg: ↑<br><br>-Lactate dehydrogenase<br>Hesperidin 100 mg/kg: ↓<br><br>-Pyruvate kinase<br>Hesperidin 100 mg/kg: ↑<br><br>-G6P<br>Hesperidin 100 mg/kg: ↓<br><br>-FBP<br>Hesperidin 100 mg/kg: ↓<br><br>-G6PD<br>Hesperidin 100 mg/kg: ↑<br><br>*Liver Glycogen and glycogen enzyme content:*<br>-Liver glycogen<br>Hesperidin 100 mg/kg: ↑<br>-Glycogen synthase<br>Hesperidin 100 mg/kg: ↑	*Hesperidin 100 mg/kg*<br><br>Body Weight gain: ↑<br>Food intake: ↓<br>Water intake: ↓<br>*Glucose tolerance test*<br>-OGGT: ↓↓ (all time points)	34

No	Compound name	Model/Dosing	Follow up period	Major outcomes*				Reference
				Blood markers	Gene expression / protein content	Tissue/cell content	Whole-animal experiments	
						-Glycogen phosphorylase<br>Hesperidin 100 mg/kg: ↓<br><br>-Muscle glycogen<br>Hesperidin 100 mg/kg: ↑<br><br>Liver weight<br>Hesperidin 100 mg/kg: ↑		
20	Hesperidin	20 ppm**	2 weeks, 3 days	N/A	N/A	N/A	Maternal Body Weight: NS (all time points)<br><br>Maternal Glycemia: ↓ (last three time points)<br><br>*Teratological parameters:*<br>-Number of foetuses: ↑<br>-Absolute foetal weight: ↑<br>-Relative foetal weight: ↑<br>-Malformations rate: ↓<br>-Resorptions rate: ↓<br>Number of implantations/litters: ↓	35
21	Sudachitin	5 mg/kg	12 weeks	-Glucose: ↓<br>-TG: ↓<br>-NEFA: ↓<br>-TCh/Ch: NS<br>-Fasting blood glucoe: ↓<br>-Insulin: ↓<br>-Leptin: ↓	GLUT-4: ↑<br>Adiponectin: ↑<br>PPARg: NS<br>aP2: NS<br>CD36: NS<br>UCP1: ↑<br>UCP2: NS<br>UCP3: ↑<br>FAS: ↓<br>ACC1: ↓<br>ACC2: ↓<br>DGAT1: NS<br>DGAT2: NS<br>SREBP1: NS<br>FDS: NS<br>SS: NS<br>HMG-R: NS<br>HMG-S: NS<br>Mttp: NS<br>LDLR: NS<br>UCP2: NS<br>ACOX: NS<br>PGC-1a: NS<br>HSL: ↑<br>CPT1a: NS<br>ATGL: NS<br>G6Pase: NS<br>PEPCK: NS<br><br>*Relative mRNA expression*	% Body fat: ↓<br>Subcutaneous fat/Body wt: ↓<br>Visceral fat/Body wt: ↓<br>Cell number: ↓	Weight: NS (all time points)<br>Food intake: NS<br>Adiponectin: ↓(last two time points)<br><br>*Glucose tolerance test*<br>-OGGT: ↓↓ (all time points)	36

No	Compound name	Model/Dosing	Follow up period	Major outcomes*				Reference
				Blood markers	Gene expression / protein content	Tissue/cell content	Whole-animal experiments	
					PGC-1a: ↑<br>Sirt1: ↑<br>NRF1: ↑<br>NRF2: ↑<br>mtTFA: ↑<br>PPARa: ↑<br>GLUT-1: ↑<br>GLUT-3: ↑<br>GLUT-4: NS<br>UCP1: NS<br>UCP2: ↑<br>UCP3: ↑<br><br>*Skeletal muscle*<br>UCP1: NS<br>UCP2: ↑<br>UCP3: NS<br><br>*Brown adipose tissue*<br>UCP1: NS<br>UCP2: NS<br>UCP3: NS<br><br>Skeletal ATP content: ↑<br>Citrate synthase: ↑			

* Changes in parameters were represented as: one arrow (↑ up or ↓ down) as a statistically significant change less than half of the value reported in no treated diabetic group, two arrows as a statistically significant change more than half of the value reported in no treated diabetic group or the same value of control group (normal value recovery), and three arrows as a statistically significant change lower/higher than control group. ** Units of dosage. ACE: angiotensin-converting enzyme; ACh: acetylcholine; SNP: sodium nitroprusside; OGTT: oral glucose tolerance test; CE: cholesteryl ester; TCh/Ch: total cholesterol; TG: triglycerides; FA: fatty acid; HDL: high-density lipoprotein; LDL: low-density lipoprotein; VLDL: very low-density lipoprotein; FFA: free fatty acids; AST: aspartate aminotransferase; ALT: alanine transaminase; ALP: alkaline phosphatase; CAT: catalase; FBP: fructose 1,6-bisphosphatase; DPP IV: dipeptidyl peptidase IV; GSP: glycosylated serum protein; GPx: glutathione peroxidase; GST: glutathione S-transferase; GK: glucokinase; G6PD: glucose-6-phosphatedehydrogenase; G6P: glucose-6-phosphatase; GPh: glycogen phosphorylase; MTP: microsomal triglyceride transfer protein; SOD: superoxide dismutase; PEPC: phosphoenolpyruvate carboxylase; PEPCK: phosphoenolpyruvate carboxykinase; PON: paraoxonase; WAT: white adipose tissue; STZ: streptozotocin; NA: nicotinamide; α-HBD: α-hydroxybutyric dehydrogenase; GK: Goto-Kakizaki rat strain; HFD: high fat diet; ob/ob: mutant ob gene model; Fgf21$^{-/-}$: negatively knockout Fgf21 gene model; ldlr$^{-/-}$: negatively knockout LDL receptor gene model; WT: wild type; AUC: area under curve; NEFA: non-esterified fatty acids; NS: no statistically significant compared with no treated diabetic group; SG: parameter was similar between genotypes/groups and treatment had no further effect (similar in groups); CN: treatment decreased/increased more than both normal and diabetic group (no modification between normal and diabetic group); TBARS: thiobarbituric acid reactive substance; HP:hyperoxides ; GR: Glutathione reductase; GSH: Glutathione; GSSG: oxidized form of glutathione; GSH/GSSG ratio: as a quantitative indicator of oxidative stress; CRP: Reactive-C Protein; LPL: Lipoprotein lipase; LCAT: lecithin cholesterol acyl transferase; NEFA: non-esterified fatty acid, N/A: Not apply.

Table S2.

Hypertension							
No.	Compound name	Model/Dosing	Follow up period	Weight	Diastolic/systolic pressure	Ions	Reference
1	Hesperidin	15-week-old Wistar and spontaneously hypertensive rats (SHR)<br><br>50 mg/kg/day	4 weeks	W: ↓	SBP: ↓<br>DBP: ND	K: ND<br>Ca: ND<br>Na: ND	37
2	Nobiletin	7 weeks old Male stroke prone spontaneously hypertensive rats (SHRSP)<br><br>20 mg/kg/day nobiletin (c1) or 40 mg/kg/day nobiletin (c2)	4 weeks	W: ↑↑	SBP: ↓↓<br>DBP: ND	K: ND<br>Ca: ND<br>Na: ND	38
3	Hesperidin	Spontaneously hypertensive rats (SHR) and normotensive Wistar-Kyoto rats (WKY)<br><br>30mg/d/kg body weight	25 weeks	W: ↑	SBP: ↓↓<br><br>DBP: ↓↓	K: ND<br>Ca: ND<br>Na: ND	39
	Glucosyl Hesperidin			W: ↑	SBP: ↓↓<br><br>DBP: ↓↓	K: ND<br>Ca: ND<br>Na: ND	
4	Apigenin	nine-week-old rats, weighing 300–325 g<br><br>1.44 mg/kg/day	6 weeks	W: ↑	SBP: ↓<br>DBP: ND	K: ND<br>Ca: ND<br>Na: ND	40
	Diosmin	9-week-old rats, weighing 300–325 g<br><br>7.16 mg/kg/day		W: ↑	SBP: ↓<br>DBP: ND	K: ND<br>Ca: ND<br>Na: ND	
5	Nobiletin	Male Sprague-Dawley rats weighing 220–250 g, induced hypertension<br><br>20 or 40 mg kg^{-1}	7 weeks	W: ↓	SBP: ↓↓<br>DBP: ↓↓<br>MAP: ↓↓<br>HR: ↓↓	K: ND<br>Ca: ND<br>Na: ND	35
6	Hesperidin/Naringenin Mixture	Isolated aorta of Wistar rats<br><br>150 mg/kg	30 days	W: ND	SBP: ↓↓<br>DBP: ↓↓<br>HR: ↓↓	K: ND<br>Ca: ↓↓<br>Na: ND	36
7	α-Glucosyl Hesperidin	Ten-week-old male Apo-E KO mice<br><br>0.5% a-glucosyl hesperidin	12 weeks	W: ND<br>TC: ↓↓	SBP: ND<br>DBP: ND	K: ND<br>Ca: ND<br>Na: ND	37
	Hesperetin	Ten-week-old male Apo-E KO mice<br><br>0.1% water-dispersible hesperetin					
8	Hesperidin	Male Sprague-Dawley rats weighing 150–180 g<br><br>20 mg/kg or 40 mg/kg	4 weeks	W: ND	SBP: ↓↓<br>DBP: ↓↓<br>MAP: ↓↓<br>HR: ↓↓	K: ND<br>Ca: ND<br>Na: ND	15

Changes in parameters were represented as: one arrow (↑ up or ↓ down) as a statistically significant change more than half of the value reported in no treated group or the same value of control group (normal value recovery), and two arrows as a statistically significant change lower/higher than control group.
ND: Undetermined¸ SBP: systolic blood pressure; DBP: diastolic blood pressure; MAP: mean arterial pressure; HR: heart rate; TC: total cholesterol; W: weight
